# Octreotide-Mediated Co-Solubilization of Paclitaxel and Docetaxel: A Novel Targeted Formulation for Synergistic Anticancer Chemotherapy

**DOI:** 10.3390/pharmaceutics18091159

**Published:** 2026-09-15

**Authors:** Ahmed A. H. Abdellatif, Hesham M. Tawfeek, Mahmoud Zaki El-Readi, Amani E. Alharbi, Hamzah M. Maswadeh, Mohammed A. Amin, Safaa Y. Eid, Abousree T. Ellethy, Bassem Refaat, Akhmed Aslam, Waleed Mohammad Altowayan, Mahmoud A. Younis

**Affiliations:** 1 Department of Pharmaceutics, College of Pharmacy, Qassim University, Qassim 51452, Saudi Arabia; msodh@qu.edu.sa (H.M.M.); m.darwish@qu.edu.sa (M.A.A.); 2 Department of Industrial Pharmacy, Faculty of Pharmacy, Assiut University, Assiut 71526, Egypt; heshamtawfeek@aun.edu.eg; 3 Faculty of Pharmacy and Drug Research, Assiut National University, New Assiut City, Assiut 11511, Egypt; 4 Department of Biochemistry, Faculty of Medicine, Umm Al-Qura University, Al Abdeyah, Makkah 24381, Saudi Arabia; mzreadi@uqu.edu.sa (M.Z.E.-R.); syeid@uqu.edu.sa (S.Y.E.); 5 Department of Pharmacology and Toxicology, College of Pharmacy, Taibah University, Madinah 42353, Saudi Arabia; aebharbi@taibahu.edu.sa; 6 Department of Basic Oral Sciences and Dental Education, College of Dentistry, Qassim University, Buraidah 51452, Saudi Arabia; aliethay@qu.edu.sa; 7 Department of Clinical Laboratory Sciences, Faculty of Applied Medical Sciences, Umm Al-Qura University, Al Abdeyah, P.O. Box 7607, Makkah 24381, Saudi Arabia; b.refaat@mnc.edu.sa (B.R.); maaslam@uqu.edu.sa (A.A.); 8 Department of Pharmacy Practice, College of Pharmacy, Qassim University, Buraydah 52571, Saudi Arabia; w.altowayan@qu.edu.sa

**Keywords:** cancer, paclitaxel, docetaxel, octreotide, clinical translation

## Abstract

**Background**: Herein, we report on the development of a novel submicron-sized formulation that utilizes the somatostatin receptor agonist, octreotide (OCT), for the solubilization and targeted delivery of paclitaxel (PTX) and docetaxel (DTX) to the cancer cells. The formulation was prepared using a fast, single-step, and scalable method, and then, it was characterized in terms of physicochemical properties and pharmacological activities. **Methods**: Formulation analyses by Electrospray Ionization Mass Spectrometry (ESI-MS) and High-Performance Liquid Chromatography (HPLC) confirmed the successful formation of the formulation and the inclusion of all of its components. **Results:** Meanwhile, the developed formulation showed acceptable physicochemical properties with no incompatibilities among its components. In addition, the prepared formulation, OCT-PTX-DTX, demonstrated superior anticancer activities against colorectal carcinoma cell lines compared to the free drugs, with minimal effects on the normal cells. Furthermore, a synergistic anticancer activity of PTX and DTX was enabled by the novel formulation, leading to cell cycle arrest at low drug concentrations. **Conclusions**: Molecular-based mechanistic investigations verified that OCT-PTX-DTX is taken up by colorectal cancer cells via somatostatin receptors 1 and 2 (SSTR1 and SSTR2), followed by the upregulation of the tumor-suppressor p53/p21 cascade, and the induced cell cycle arrest via suppression of the cell cycle drivers CDK2 and Cyclin A. The formulation reported herein is promising for targeted and biotolerable anticancer chemotherapy compared to the commercially available taxane formulations based on surfactants or organic solvents.

## 1. Introduction

Cancer has been a leading health threat that returned to the forefront in the post COVID-19 era, where the incidence and mortality figures are expected to boom due to the disturbed routine screening during the pandemic [1,2,3]. While new drug development requires huge funds and a long time, improving the efficacy of existing treatments may be an alternative rational strategy to cope with the expected surge [4].

Chemotherapy is the major therapeutic line for non-resectable and metastatic cancers [5]. Taxanes represent a potent class of anticancer chemotherapies, derived from the yew tree (genus *Taxus*), that have been approved since the 1990s for use in various cancers, including ovarian, breast, prostate, lung, and colon cancers. They act by disrupting microtubules, essential for cell division, leading to mitotic arrest [6,7]. Paclitaxel (PTX) and docetaxel (DTX) are the key members of the taxane family. However, their clinical efficacy is limited by multiple challenges, including their poor aqueous solubility leading to limited bioavailability, as well as their detrimental impacts on healthy cells [8]. Tremendous efforts have been exerted to tackle such challenges. For instance, Cremophor EL (polyoxyethylated castor oil) and dehydrated ethanol are commercially used to emulsify PTX for parenteral administration. Similarly, Tween 80 and ethanol are used to solubilize DTX. Nevertheless, such methodologies raise biosafety concerns owing to toxic and allergic effects induced by the use of high concentrations of surfactants or organic solvents [9].

Active targeting has been extensively reported in the literature in an attempt to exploit the overexpression of certain receptors on the surface of cancer cells in improving the selectivity of chemotherapies and reducing their off-target effects [10]. Octreotide (OCT) is a synthetic octapeptide-based agonist for somatostatin receptors (SSTRs) that is used in the treatment of acromegaly and neuroendocrine tumors, as well as diarrhea associated with metastatic carcinoid tumors and vasoactive intestinal peptide secreting tumors [11,12,13]. Moreover, thanks to the overexpression of SSTRs in diverse tumors, OCT has been recruited as a tumor-specific targeting ligand [14,15]. Nonetheless, some recent reports have criticized ligand-based active targeting due to the increased complexity of the drug delivery systems, which subtracts from their scalability and clinical translatability [16].

In the present study, we developed a novel formulation, referred to as OCT-PTX-DTX, that harnesses OCT in simultaneously solubilizing a combination of PTX and DTX, while attaining their selective delivery to the cancer cells, using a single-step preparation method. Thus, the composition of the delivery system is kept simple for translational purposes. Moreover, the submicron particle size of the formulation assists in improving the solubility, dissolution, and cellular uptake of the drug payload. The developed formulation was evaluated for both physicochemical properties and pharmacological effects in various cancerous and normal cells. Furthermore, the formulation enabled the synergism between the two drugs in question to maximize the therapeutic efficiency at lower drug concentrations and lower side effects compared to the commercially available solubilization methods.

## 2. Materials and Methods

### 2.1. Materials

Paclitaxel (purity 99.97%) was purchased from MedChemExpress (Monmouth Junction, NJ, USA). Docetaxel (Purity 98%) was purchased from BioSynth UK (Berkshire, UK). Octreotide acetate (purity 99.32%) was purchased from Santa Cruz Biotechnology, Inc. (Dallas, TX, USA). Acetonitrile, methanol, and formic acid (LC/MS grade) were obtained from ThermoFisher Scientific, Waltham, MA, USA. The MTT Assay Kit was purchased from Abcam (Cambridge, UK). PBS, RNase, and propidium iodide were purchased from Sigma-Aldrich (MO, St. Louis, USA). RNeasy Mini Kit was supplied by Qiagen (Hilden, Germany). The High-Capacity cDNA Reverse Transcription Kit was purchased from Applied Biosystems (Carlsbad, CA, USA). All chemicals were of analytical grade without additional processing.

### 2.2. Cell Culture

The human colorectal cancer cell lines, HT29, SW480, and SW620, as well as the normal human fibroblast cell line, MRC-5, were obtained from the American Type Culture Collection (ATCC) (Manassas, VA, USA) and were handled according to the manufacturer’s guidelines. The cells were cultivated in Dulbecco’s Modified Eagle Medium (DMEM) (Gibco, Thermo Fisher Scientific, Waltham, MA, USA) supplemented with 10% *v*/*v* Fetal Bovine Serum (FBS), 100 U/mL penicillin, and 100 U/mL streptomycin. The cells were incubated at 37 °C, in an atmosphere of CO_2_ (5%) and humidity (95%).

### 2.3. Preparation of OCT-PTX-DTX Formulation

DTX, PTX, and OCT were dissolved in a mixture of ethanol and double-distilled water (1:1 *v*/*v*) at concentrations of 1.3 mg/mL, 0.9 mg/mL, and 2.2 mg/mL, respectively. The dispersion was kept under continuous stirring until the complete evaporation of the organic solvent. The resultant OCT-PTX-DTX colloidal dispersion was kept at 4 °C.

### 2.4. Characterization of OCT-PTX-DTX Formulation

#### 2.4.1. Compatibility of Formulation Components

Differential Scanning Calorimetry (DSC) was used to investigate the compatibility of the various components of the formulation as described previously [17]. Briefly, 5 mg samples were introduced into a 50 µL aluminum pan, press-sealed with an aluminum cover, and scanned in a Shimadzu DSC-50 apparatus (Shimadzu, Kyoto, Japan) up to 300 °C, at a rate of 10 °C/min and nitrogen flow of 25 mL/min. The obtained thermogram was visualized and analyzed using the built-in TA-50 software.

#### 2.4.2. Electrospray Ionization Mass Spectrometry (ESI-MS)

In order to confirm the identity and inclusion of all components in the developed formulation, high-resolution ESI-MS analyses were performed on a Thermo Scientific Q Exactive hybrid quadrupole-Orbitrap mass spectrometer with a ZipChip system, equipped with an electrospray ionization source (ThermoFisher Scientific, USA). Samples were dissolved in the following solvent systems: 100% methanol with 0.1% formic acid for PTX and DTX, 50:50 (*v*/*v*) methanol/water with 0.1% formic acid for OCT, and 70:30 (*v*/*v*) methanol/water with 0.1% formic acid for OCT-PTX-DTX. Samples were infused directly into the ion source at a flow rate of 10 µL/min using a syringe pump. The instrument was operated in the positive ion mode over a mass-to-charge ratio (*m*/*z*) range of 100–1500, capillary voltage of 3.5 KV, source temperature of 250 °C, desolvation gas (N_2_) flow of 40 Arb, and S-lens RF level of 50. Data acquisition and processing were performed using Xcalibur v4.2 software (ThermoFisher Scientific, USA) [18,19].

#### 2.4.3. High-Performance Liquid Chromatography (HPLC)

The components of the OCT-PTX-DTX formulation were resolved and detected by HPLC on a reversed-phase Zorbax Eclipse XDB C18 column (Rapid Resolution 4.6 × 150 mm, 5 µm) (Agilent Technologies, Santa Clara, CA, USA), using an Agilent Technologies 1200 Series, G1315D apparatus (Agilent Technologies, USA). PTX and DTX were eluted through isocratic elution using a mobile phase composed of water and acetonitrile (70:30 *v*/*v*, respectively). Meanwhile, OCT was eluted through isocratic elution using a mobile phase composed of 20 mM phosphate buffer (pH 7.4) and acetonitrile (65:35 *v*/*v*, respectively). An autosampler was used to inject samples at a 20 µL volume and 25 °C, and the flow rate was kept at 1 mL/min. A diode array detector (DAD) was used to detect PTX and DTX at a wavelength of 232 nm and OCT at a wavelength of 210 nm [20,21].

#### 2.4.4. Particle Size and Particle Size Distribution

The particle diameter as well as the particle size distribution of OCT-PTX-DTX were assessed using the SALD-2300 Laser Diffraction Particle Size Analyzer (Shimadzu, Japan).

#### 2.4.5. Particle Morphology

The morphology of OCT-PTX-DTX particles was visualized by transmission electron microscopy (TEM) as described previously [22]. Samples of OCT-PTX-DTX were air-dried overnight onto a copper conductive tape surface. Then, a TEM microscope (JEM-1230, JEOL, Akishima (Tokyo), Japan) was used to investigate the samples at a magnification power of 10–100 k and an accelerating voltage of 100 KV.

### 2.5. Assessment of Cell Viability

The 3-(4,5-Dimethylthiazol-2-yl)-2,5-Diphenyltetrazolium Bromide (MTT) assay was used to determine cell viability as reported previously [23]. The cells in question were subcultured and seeded at a density of ${1\times 10}^{4}$ cells/well in 96-well plates and incubated for 24 h. Then, the cells were subjected to 0.1–200 nM of the tested samples and incubated for an extra 24 h. Subsequently, 20 μL of MTT reagent in PBS (-) (2 mg/mL) was mixed with the culture medium to a final concentration of 0.5 mg/mL and incubated for 3 h at 37 °C. The supernatants were carefully aspirated, and the reaction product was dissolved in 100 μL of DMSO. Finally, the optical density (OD) was measured spectrophotometrically at 570 nm, using DMSO as a blank (GraphPad Prism Version 9). The cell viability of the treated samples was expressed as a percentage of the values for vehicle-treated cells, which were considered to have a cell viability of 100%.

### 2.6. Analysis of Interaction Between PTX and DTX

The viabilities of cells treated with PTX, DTX, OCT, and OCT-PTX-DTX were individually assessed by MTT assay as described above, where OCT and OCT-PTX-DTX were used in aqueous solution and colloidal dispersion forms, respectively. Meanwhile, PTX and DTX were dissolved in DMSO before mixing with the cell culture media to result in a final DMSO concentration of <0.01%, where DMSO did not show any impact on the cell viability [24]. The interactive pharmacological effects of PTX and DTX in the OCT-PTX-DTX formulation were verified through the calculation of combination indices (CI) using Equation (1) [25].

(1) CI = (C_PTX,50_/PTX_IC50_) + (C_DTX, 50_/DTX_IC50_)

where CI is the combination index, C_PTX,50_ and C_DTX,50_ represent the half-maximal effective concentration (IC_50_) values for the two drugs in combination (formulation), and PTX_IC50_ and DTX_IC50_ represent the IC_50_ values of each drug in a singular form. CI < 1 suggests synergism, CI = 1 suggests addition, while CI > 1 suggests antagonism. For further verification of the interactive relationship, the isobologram method was used [26,27].

### 2.7. Cell Cycle Analysis

The cells in question were incubated with the tested samples at 37 °C for 6 h. Then, the cells were double-washed with ice-cold PBS (-), harvested, and centrifuged at 1000 rpm for 5 min. The cells were subsequently fixed with 70% ethanol for 6 h at 4 °C. Lastly, the cells were double-washed with cold PBS (-), resuspended in 0.1 mg/mL RNase, stained with 40 mg/mL propidium iodide (PI), and analyzed with flow cytometry using FACS Calibur apparatus (Beckman Coulter, Brea, CA, USA). The distributions of the cell cycle were analyzed and presented in a FACS histogram [28].

### 2.8. Quantitative Real-Time Polymerase Chain Reaction (qRT-PCR)

Following the manufacturer’s instructions, total RNA was extracted from both treated and untreated cells using the RNeasy Mini Kit (Qiagen, Germany). A NanoDrop spectrophotometer (Thermo Fisher Scientific, Waltham, MA, USA) was used to measure the extracted RNA’s concentration and purity; A260/A280 ratios between 1.8 and 2.1 were deemed acceptable. A high-capacity cDNA reverse transcription kit (Applied Biosystems, Carlsbad, CA, USA) was then used to reverse-transcribe 1 μg of total RNA into complementary DNA (cDNA) in a final reaction volume of 20 μL. Fast Real-Time PCR System (Applied Biosystems, Carlsbad, CA, USA) was used to measure the mRNA expression levels of SSTR1, SSTR2, p53, p21, CDK2, and Cyclin A in a reaction volume of 20 μL, using the oligonucleotide qPCR primers listed in Table S1. GAPDH was used as the internal housekeeping gene for normalization when calculating relative gene expression levels using the 2^−ΔΔCt^ method [29]. Every experiment was carried out in triplicate (*n* = 3).

### 2.9. Statistical Analysis

The SPSS 26.0 software (IBM SPSS Statistics, Armonk, NY, USA) was used for statistical analyses in this study. For comparing more than two groups, one-way analysis of variance (ANOVA) and Tukey’s post hoc tests were used. A confidence level of 95% was adopted. * *p* < 0.05, ** *p* < 0.01, and *** *p* < 0.001 were assumed to be statistically significant.

## 3. Results

### 3.1. Preparation and Characterization of OCT-PTX-DTX Formulation

**Figure 1:**
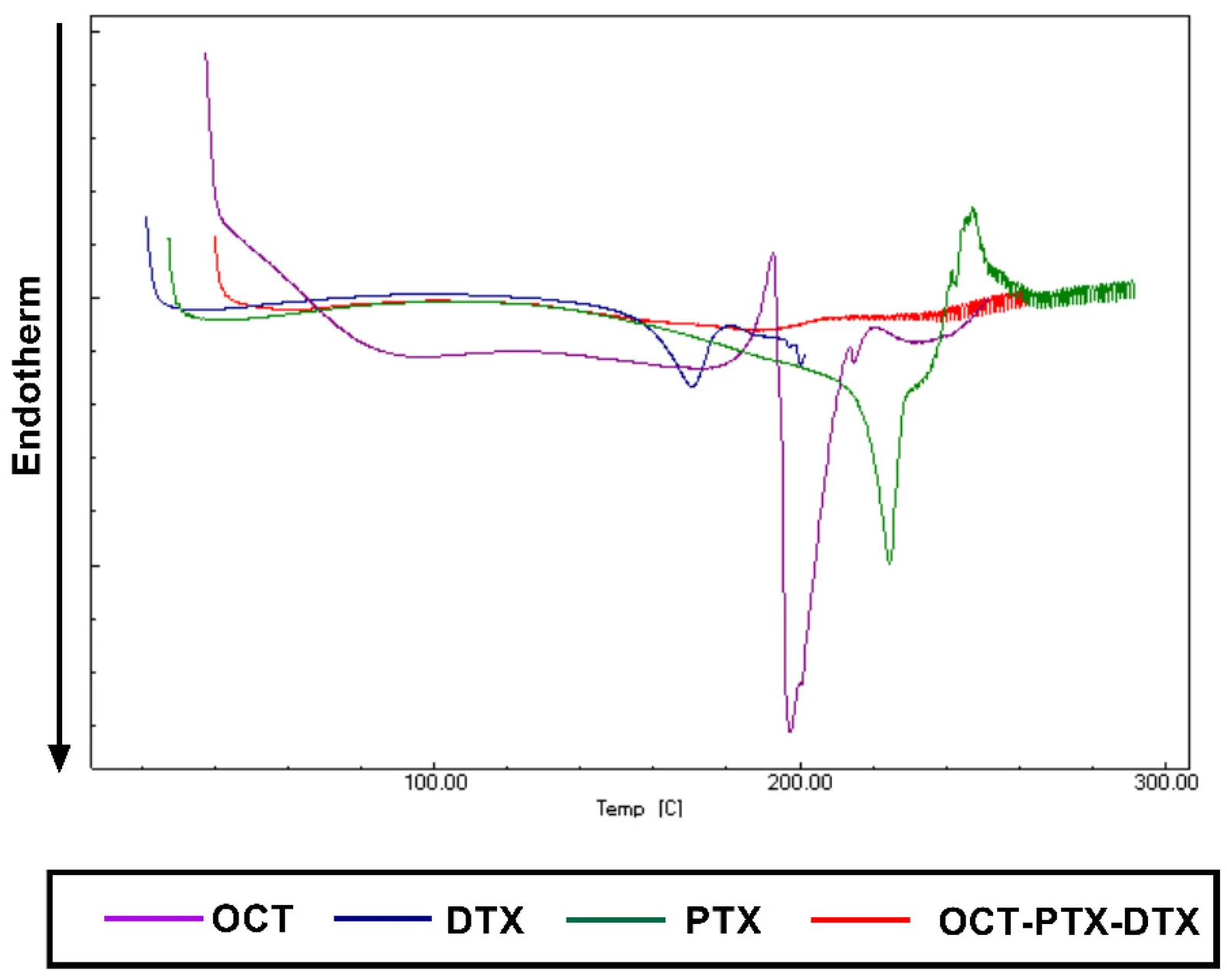
DSC thermograms of OCT, PTX, DTX, and OCT-PTX-DTX formulation.

The novel OCT-PTX-DTX formulation was successfully prepared, resulting in a homogeneous colloidal solution without visible particle aggregates or sediments, suggesting an initial physical stability. The compatibility of the various components in the formulation was investigated by DSC. Pure DTX and OCT demonstrated sharp endothermic peaks at 160–180 °C and 180–200 °C, respectively. Meanwhile, pure PTX showed an endothermic peak at 210–230 °C, followed by an exothermic peak at 240–260 °C. On the other hand, OCT-PTX-DTX formulation did not show any peaks within the investigated temperature range (Figure 1).

In addition, the formulation components were verified and characterized by ESI-MS. The analysis of mass spectra successfully identified the distinct mass-to-charge ratios (*m*/*z*) for the key components: DTX at *m*/*z* = 807.9, PTX at *m*/*z* = 854.11, and OCT at *m*/*z* = 1019.49. Furthermore, the retention time analysis for PTX (9.85 min) and OCT (6.76 min) validated their purity and chromatographic separation within the formulation matrix. Notably, the data also revealed approximately 25% transition in the PTX peak and disappearance of the DTX peak in the final formulation (Figure S1). Furthermore, HPLC analysis of the final OCT-PTX-DTX formulation confirmed the existence of the three components (Figure S2).

**Figure 2:**
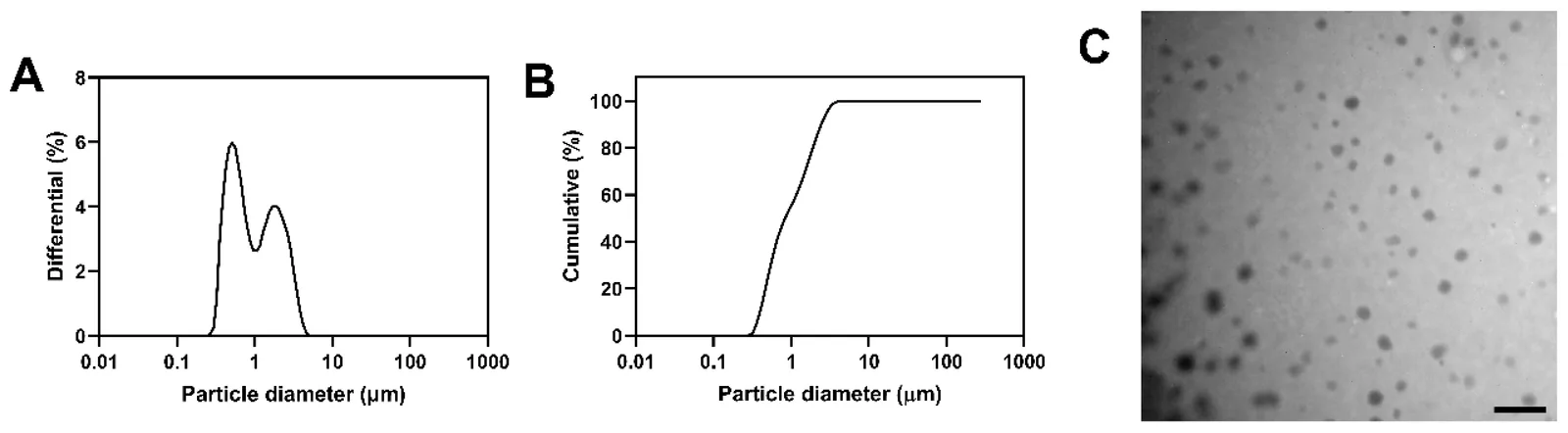
Analysis of the differential (**A**) and cumulative (**B**) particle size distributions of OCT-PTX-DTX as well as the particle morphology (**C**). The particle size distribution was analyzed using a Shimadzu laser diffraction particle size analyzer. Meanwhile, the particle morphology was visualized by TEM (JEM-1230, JEOL, Japan). The scale bar represents 500 nm.

Analysis of particle size distribution revealed a bimodal differential distribution with peaks around 0.5 µm and 1.6 µm, respectively (Figure 2A). The cumulative particle size distribution revealed a median particle diameter of 0.837 µm (Figure 2B, Table S2). Visualization of particle morphology by TEM showed homogeneous spherical particles with an average diameter around 200 nm (Figure 2C).

### 3.2. OCT-PTX-DTX Formulation Demonstrates Selective Cytotoxicity to Cancer Cells

**Figure 3:**
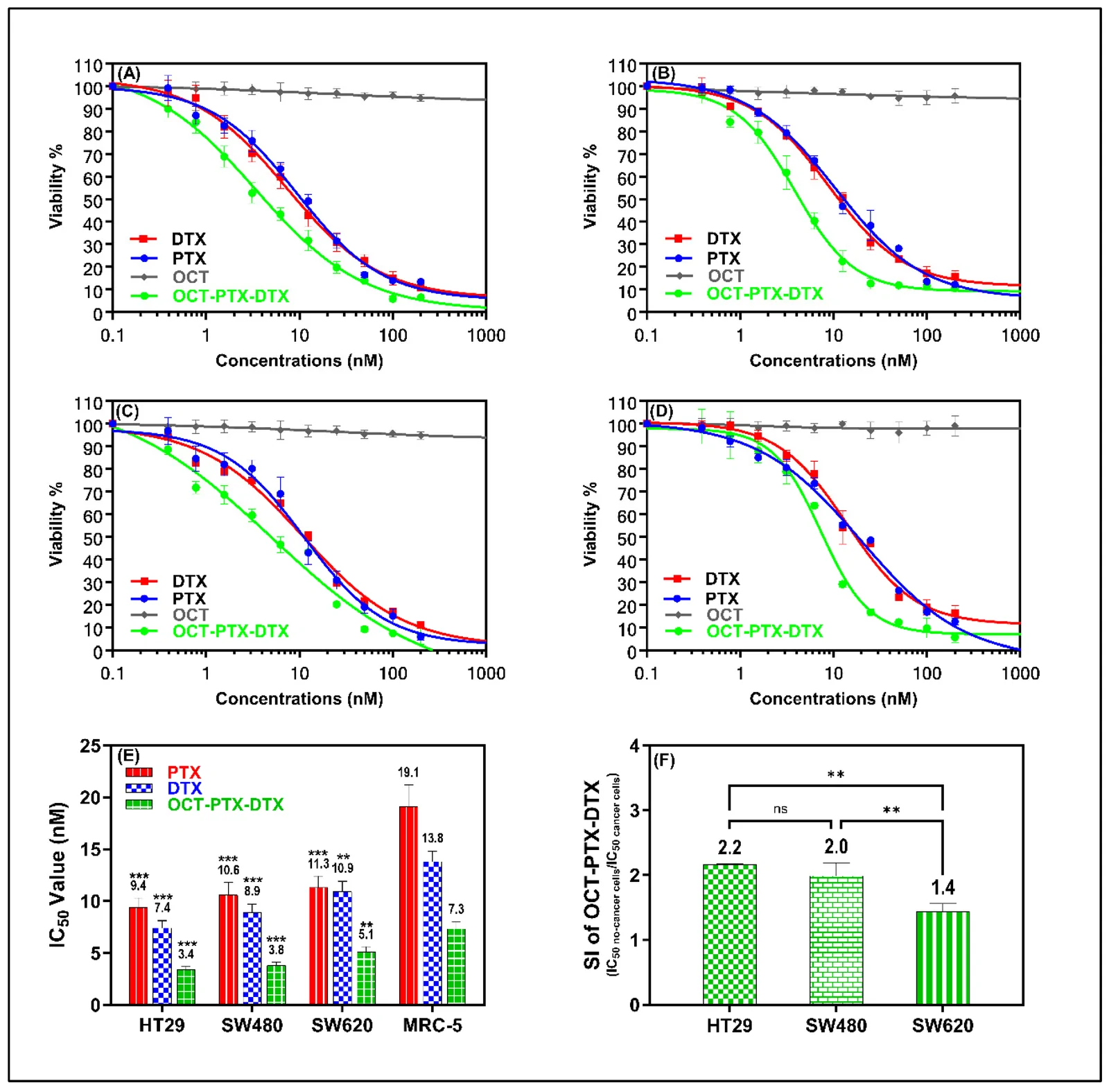
OCT-PTX-DTX formulation demonstrates selective cytotoxicity to cancerous cells. The dose–response curves of PTX, DTX, OCT, and OCT-PTX-DTX on the cell viability of colon cancer cells, HT29 (**A**), SW480 (**B**), and SW620 (**C**), in comparison with normal cells, MRC-5 (**D**), were constructed. The MTT assay was used to assess the viability of the various cell lines after 24 h of their treatment with the investigated samples at concentrations ranging from 0.1 to 200 nM. (**E**) IC_50_ values of OCT-PTX-DTX in the various cell lines in question. ** *p* < 0.01 and *** *p* < 0.001 versus MRC-5 cells. (**F**) Selectivity index (SI) values of OCT-PTX-DTX to the various investigated cancerous cell lines. SI was calculated by comparing individual IC_50_ values of OCT-PTX-DTX formulation in the normal cells versus their cancerous counterparts under the same conditions. ns, statistically non-significant; ** *p* < 0.01. *N* = 3; the average of each measure is recorded with ±SD.

Cytotoxic effects of free PTX, free DTX, OCT, and OCT-PTX-DTX were assessed on three different human colorectal carcinoma cell lines: HT29 (Figure 3A), SW480 (Figure 3B), and SW620 (Figure 3C) in comparison with the human fibroblast cell line, MRC-5, as a normal cell model (Figure 3D). The tested samples were screened within a broad concentration range of 0.1–200 nM, and dose–response survival curves were constructed accordingly. Interestingly, OCT did not show any impact on cell viability at all the investigated concentrations. The results revealed that OCT-PTX-DTX had a superior performance on all cells in question compared to the free drugs. Based on IC_50_ values, HT29 cells were the most sensitive cells to the treatment (IC_50_ = 3.4 nM), followed by SW480 (IC_50_ = 3.8 nM) and SW620 cells (IC_50_ = 5.4 nM), respectively (Figure 3E). On the other hand, the formulated system resulted in a significantly lower cytotoxicity to the normal cells, MRC-5, compared to the cancerous cells in question, as suggested from the selectivity index values, in which the IC_50_ values in the normal cells were compared to the IC_50_ values in their cancerous counterparts (Figure 3F).

### 3.3. Synergistic Anticancer Effects of the Formulated System

**Figure 4:**
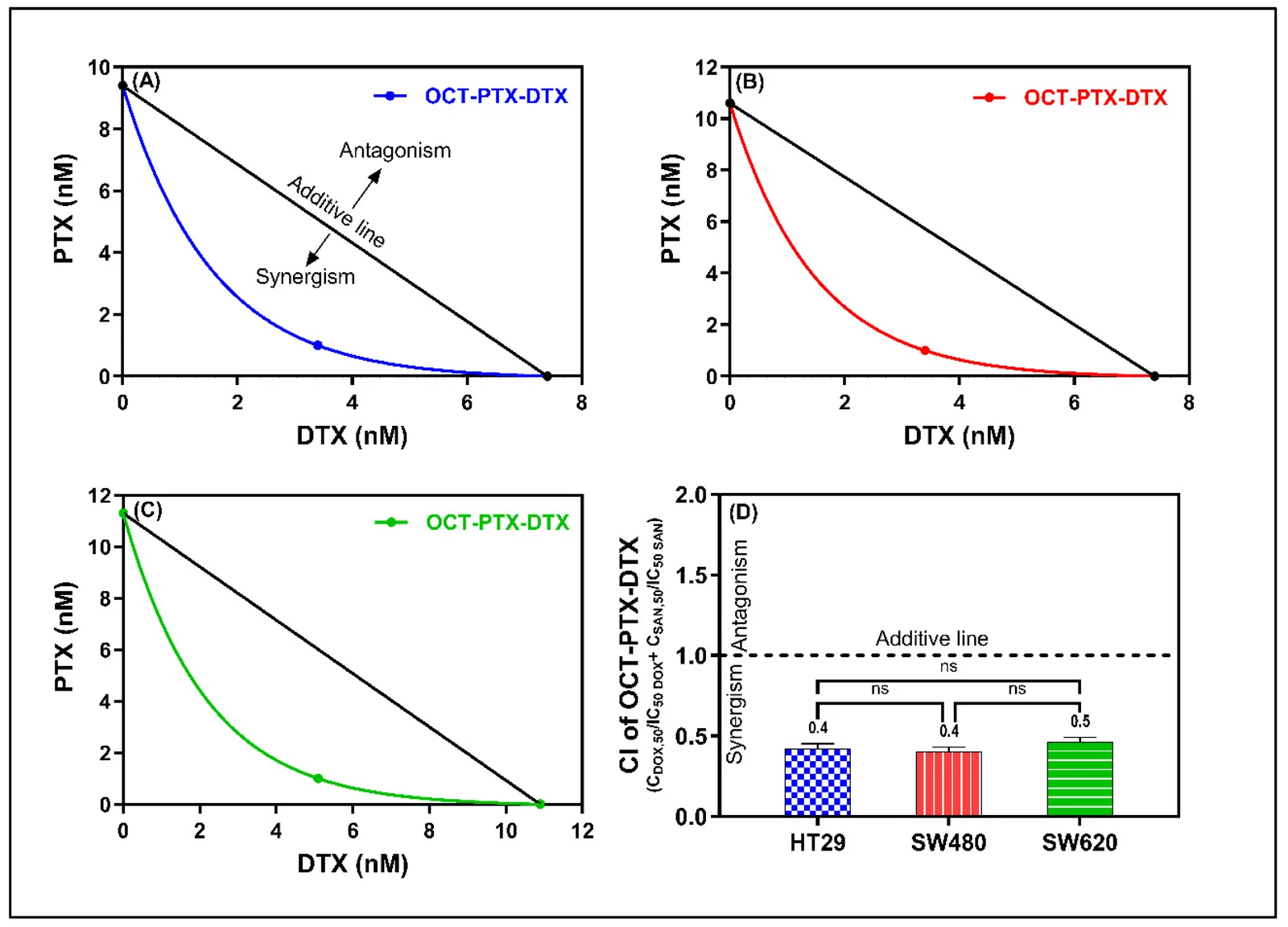
The novel OCT-PTX-DTX formulation renders a synergistic anticancer activity of PTX and DTX against the colon cancer cells. (**A**–**C**) Isobolograms of PTX and DTX in HT29 cells, SW480 cells, and SW620 cells, respectively. Isobolograms were constructed using IC_50_ values. The average of 3 independent experiments was considered in calculations. The straight line represents the additive effect of the two drugs in question. (**D**) The combination index (CI) values in the various investigated cells. N = 3; the average of each measure is recorded with ±SD.

In order to evaluate the synergistic interaction of PTX and DTX in the novel OCT-PTX-DTX formulation, the isobologram and combination index (CI) analyses were applied. Isobologram plots showed a downward deviation from the additive line of the two drugs in question (Figure 4A–C). Meanwhile, CI values were 0.4 in the case of either HT29 or SW480 cells and 0.5 in the case of SW620 cells (Figure 4D).

### 3.4. OCT-PTX-DTX Formulation Efficiently Inhibits Cancer Cell Cycle

**Figure 5:**
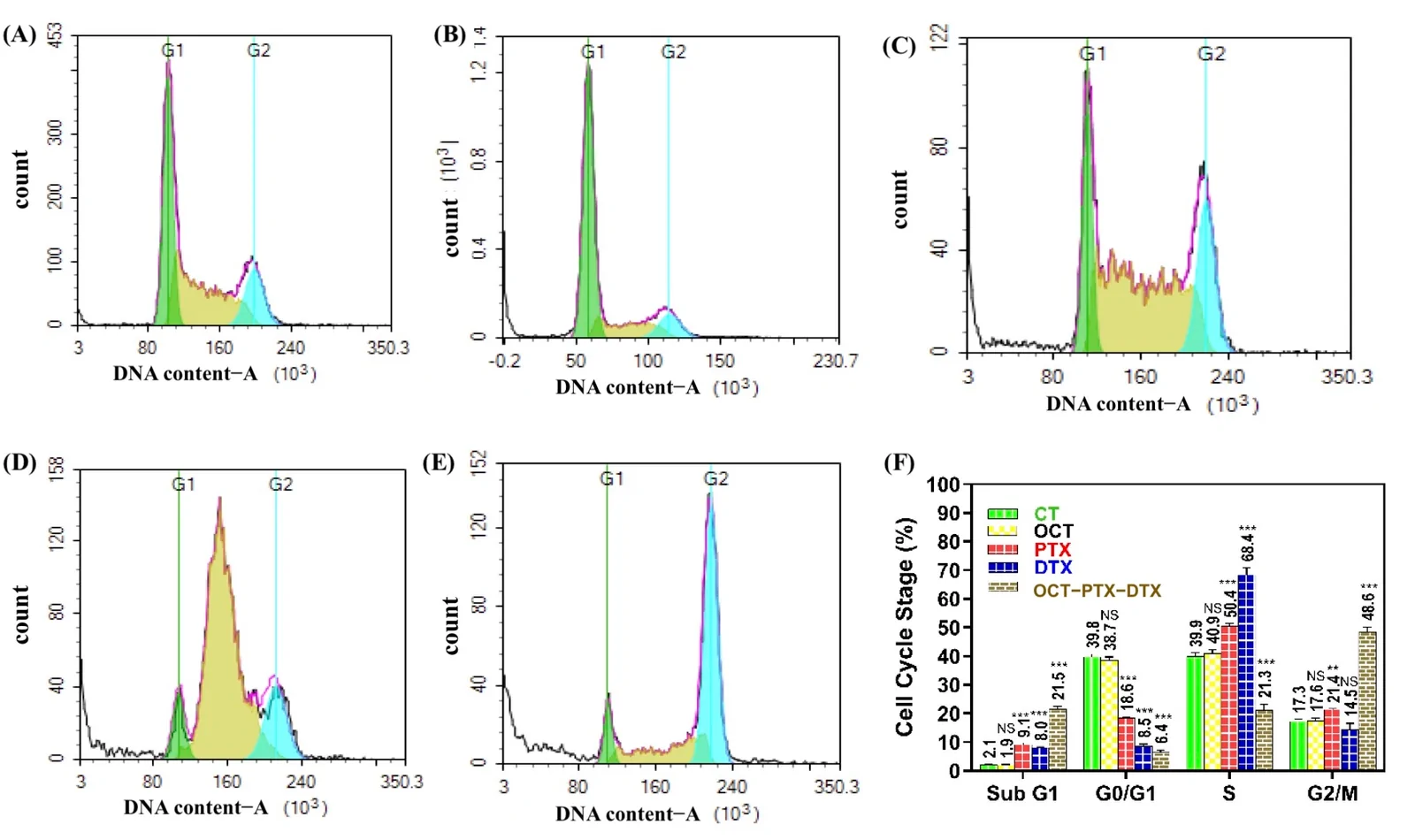
The impact of various treatments on the cell cycle phases of SW480 cells. Untreated cells (negative control, Ctrl) (**A**) or cells treated with OCT (**B**), PTX (**C**), DTX (**D**), and OCT-PTX-DTX (**E**) were fixed, stained with PI, and analyzed by flow cytometry. The percentages of cells in each cycle phase were compared (**F**). NS, statistically non-significant, ** *p* < 0.01, *** *p* < 0.001 versus the negative control. The average of 3 independent experiments was considered in calculations and presented with ±standard deviation.

**Figure 6:**
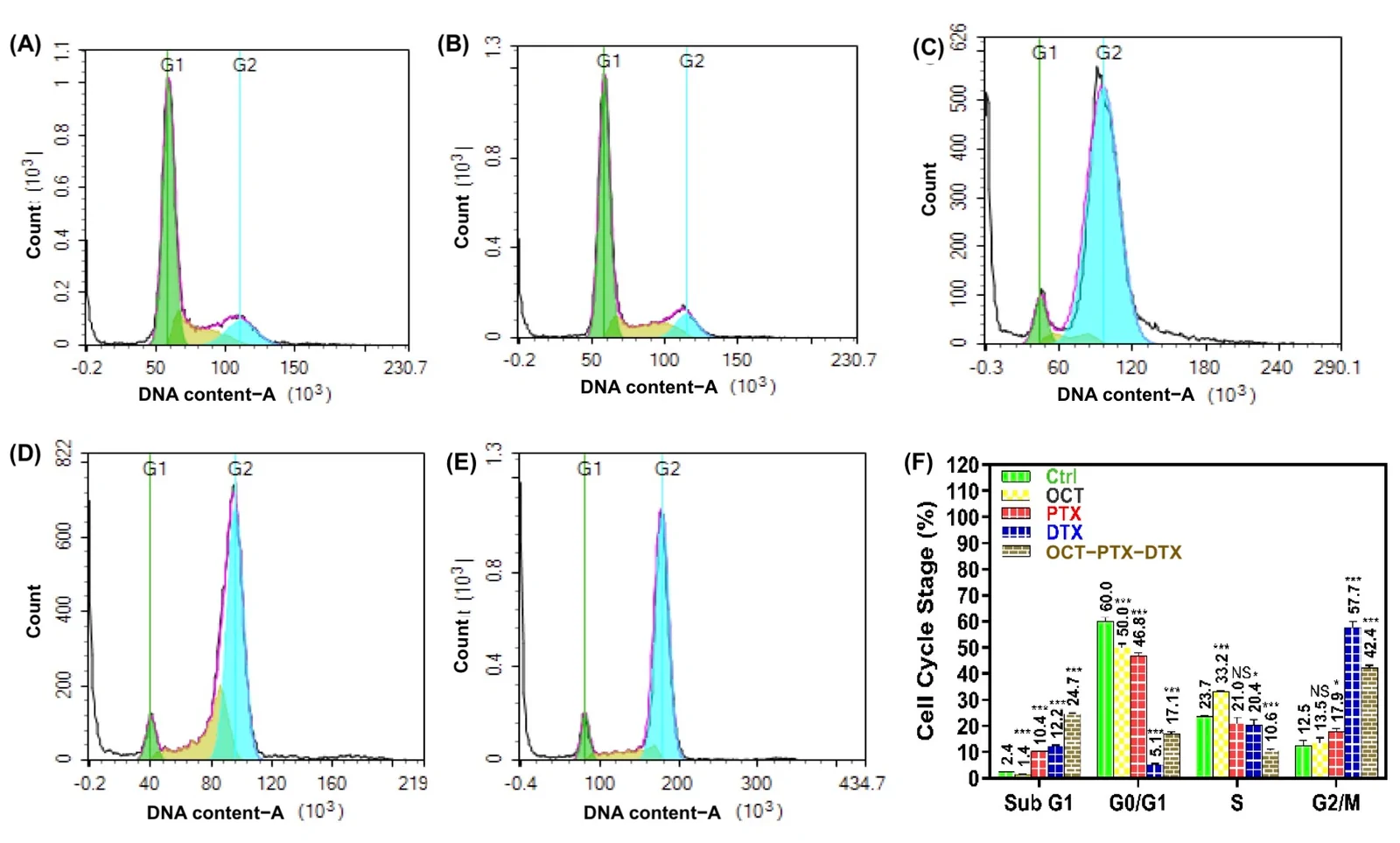
The impact of various treatments on the cell cycle phases of HT29 cells. Untreated cells (**A**) or cells treated with OCT (**B**), PTX (**C**), DTX (**D**), and OCT-PTX-DTX (**E**) were fixed, stained with PI, and analyzed by flow cytometry. The percentages of cells in each cycle phase were compared (**F**). NS, statistically non-significant; * *p* < 0.05; *** *p* < 0.001 versus the negative control. The average of 3 independent experiments was considered in calculations and presented with ±standard deviation.

Cell cycle analysis was performed for further verification and mechanistic investigation of the anticancer effects of the OCT-PTX-DTX formulation on SW480 cells. OCT showed a negligible impact on the cell cycle, which was nearly identical to that of the negative control. Meanwhile, the novel OCT-PTX-DTX formulation significantly increased the percentage of cells in the sub-G1 phase (21.5%) compared to the individual drugs (9.1% and 8% for PTX and DTX, respectively). In addition, OCT-PTX-DTX significantly reduced the percentage of cells in G0/G1 and S phases. On the other hand, the formulation resulted in a significant increase in the percentage of cells in the G2/M phase compared to all investigated samples (Figure 5A–F). Furthermore, in order to confirm the robustness of such anticancer effects, the above experiment was reproduced in a different colon cancer cell line, HT29. The results revealed a similar pattern to that obtained with SW480 cells, where OCT-PTX-DTX increased the percentage of cells in sub-G1 and G2/M phases while decreasing those in G0/G1 and S phases (Figure 6A–F).

### 3.5. Mechanistic Insights on the Developed Formulation

**Figure 7:**
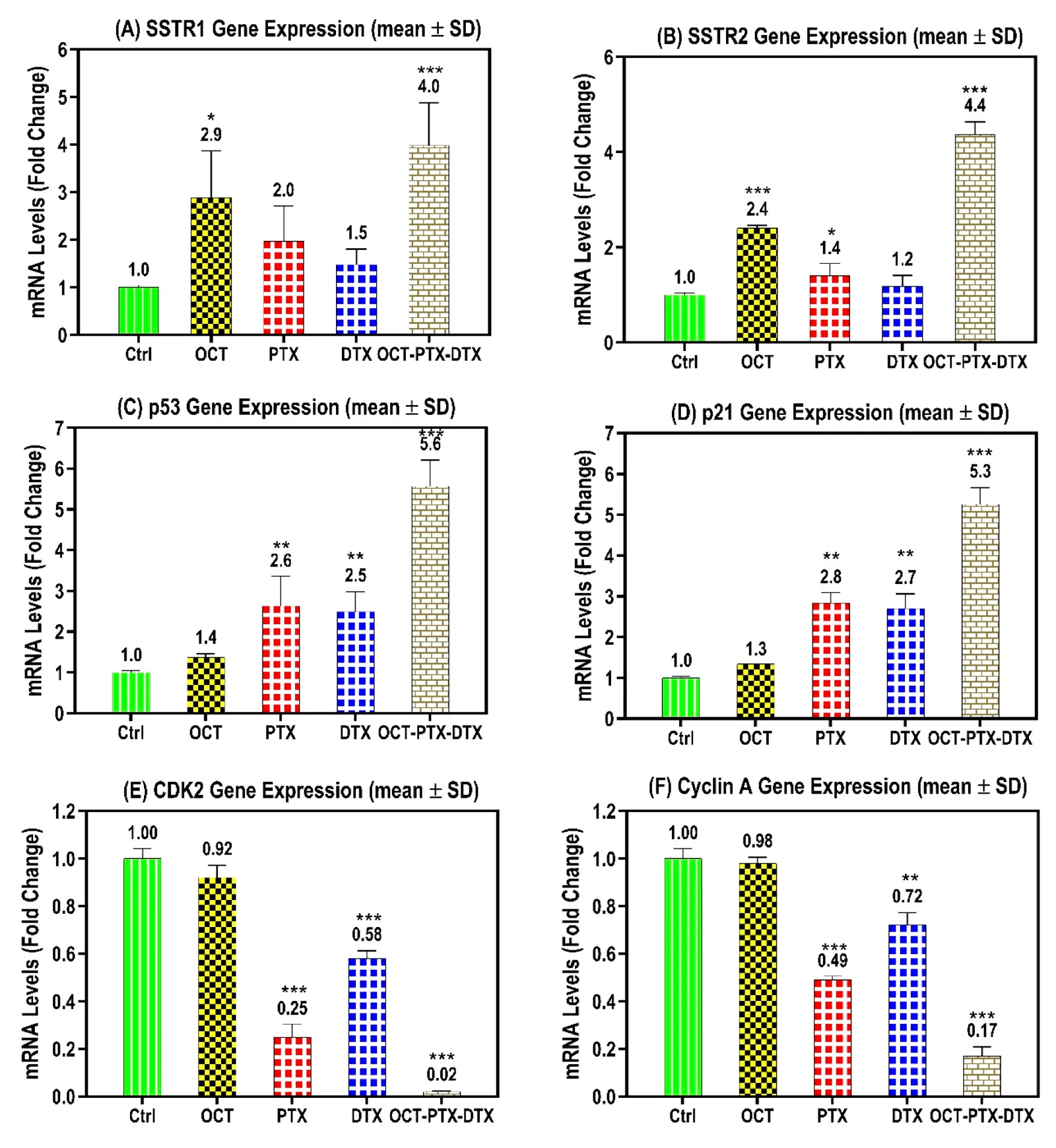
Mapping the gene expression profiles of the various relevant genes in SW480 cells following treatment with control (Ctrl), octreotide (OCT), paclitaxel (PTX), docetaxel (DTX), and the combined targeted formulation (OCT-PTX-DTX). qRT-PCR analysis was performed, and the fold change in mRNA levels of the genes in question is presented relative to the negative control. (**A**) SSTR1. (**B**) SSTR2. (**C**) p53. (**D**) p21. (**E**) CDK2. (**F**) Cyclin A. Data are presented as mean ± SD (n=3) as * p<0.05, ** p<0.01, and *** p<0.001 compared to the control cells. The targeted OCT-PTX-DTX group exhibited significant synergistic upregulation of tumor suppressors (p53/p21) and somatostatin receptors (SSTR1/2), alongside a near-complete suppression of the cell cycle drivers CDK2 and Cyclin A.

The above results prompted us to attempt further in-depth understanding of the molecular basis of the cellular actions of OCT-PTX-DTX formulation. Therefore, we mapped the gene expression profiles of the various relevant genes in the SW480 cells post-treatment with OCT-PTX-DTX or its individual components. In terms of modifying the somatostatin receptor expression, OCT-PTX-DTX formulation proved to be more effective. Somatostatin receptor 1 (SSTR1) and somatostatin receptor 2 (SSTR2) mRNA levels were significantly higher than the control, increasing to 4.0-fold (*p* < 0.001) and 4.4-fold (*p* < 0.001), respectively (Figure 7A,B). This upregulation was significantly greater than that seen with OCT, PTX, or DTX alone, suggesting an improved receptor-mediated internalization of the targeted delivery system. In addition, the p53-dependent apoptotic pathway was robustly activated by the OCT-PTX-DTX treatment, as evidenced by a 5.6-fold (*p* < 0.001) increase in p53 expression, which nearly doubled the effect of PTX alone (2.6-fold). As a result, the downstream effector p21 increased by 5.3-fold (*p* < 0.001) (Figure 7C,D). A high degree of cellular stress brought on by DNA damage, which results in programmed cell death, is indicated by this strong induction of the p53-p21 axis. In the treated cells, CDK2 mRNA levels were almost eliminated, falling to 0.02-fold (98% reduction, *p* < 0.001) (Figure 7E). The expression of cyclin A was significantly reduced to 0.17-fold (*p* < 0.001) (Figure 7F). The formulation’s ability to cause G1/S or G2/M phase arrest by blocking the Cyclin A/CDK2 complex is demonstrated by the dramatic drop in these markers, particularly when contrasted with the partial effects of free PTX or DTX.

## 4. Discussion

Combinational chemotherapy has been increasingly adopted in the clinical management of a wide variety of cancers to integrate the unique benefits of diverse drugs, impart synergism, maximize the therapeutic outcomes, and overcome chemoresistance [30,31]. There has been growing evidence suggesting promising therapeutic outcomes upon combining taxanes, PTX, and DTX in diverse types of malignancies, including breast, ovarian, and colon cancers [32,33,34,35]. However, the efficacy of such combinations is usually affected by the rate of their delivery to the site of action as well as the sequence of treatment, which affects their pharmacokinetic performance [36,37]. In addition, the poor aqueous solubility of taxanes is limiting their bioavailability [38]. To tackle these challenges, we developed a novel formulation combining both drugs in question, thus ensuring their simultaneous delivery to the cancer cells on the molecular level and overcoming any discrepancies arising from different rates of delivery or pharmacokinetic profiles.

**Figure 8:**
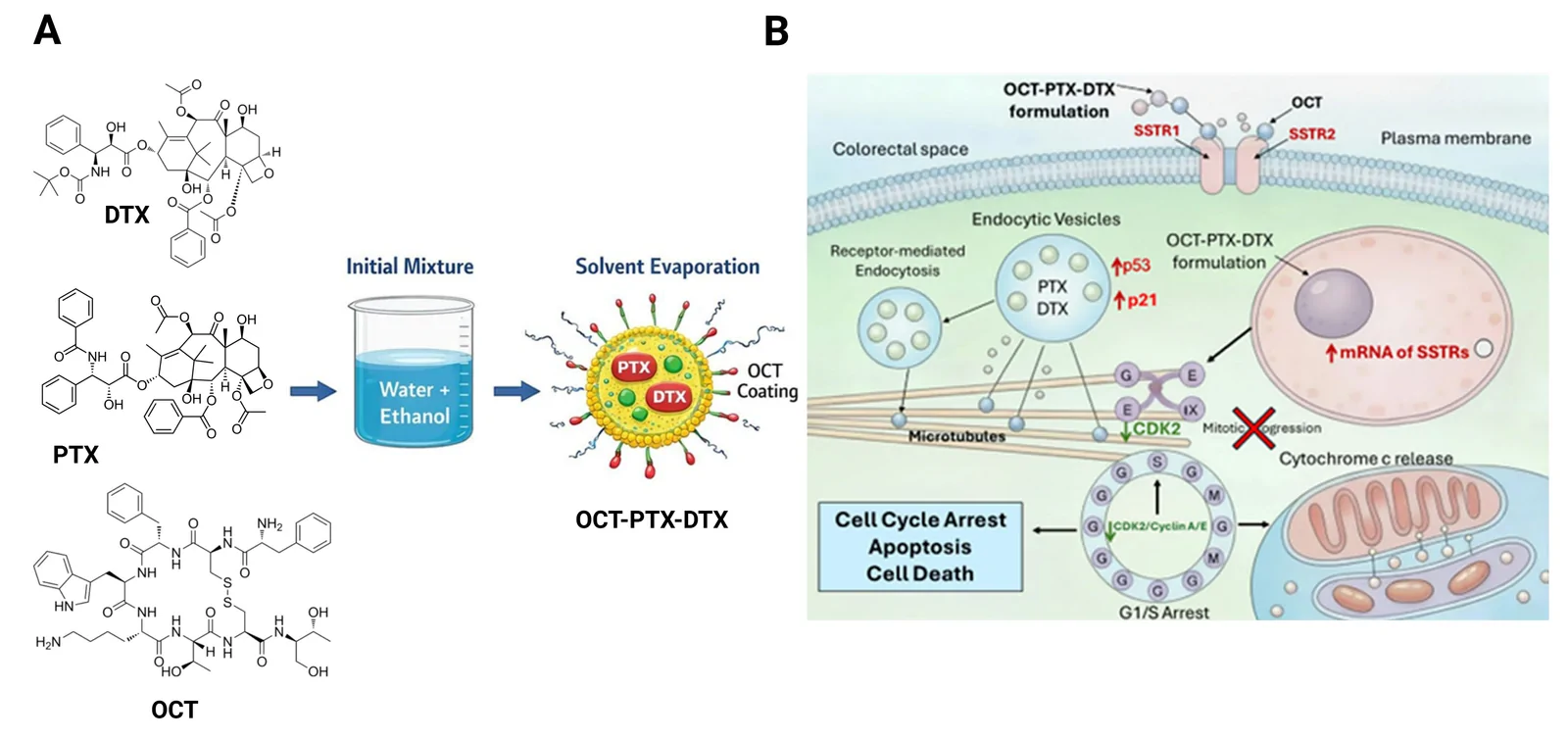
Proposed molecular mechanisms of OCT-PTX-DTX formation and cellular actions. (**A**) Formation of OCT-PTX-DTX through solvent evaporation method. OCT, PTX and DTX are initially dissolved in a mixture of ethanol and water. Upon evaporation of the organic solvent, the poorly water-soluble drugs in question become dispersed into the water-soluble matrix of OCT, which renders their solubilization. (**B**) Cellular actions of OCT-PTX-DTX. OCT mediates the internalization of the formulation via receptor-mediated endocytosis through the overexpressed SSTRs. The binding of OCT to SSTRs triggers further upregulation of SSTRs through a positive feedback mechanism, allowing further internalization of OCT-PTX-DTX particles. The internalized formulation triggers the upregulation of tumor suppressors (p53/p21) alongside a near-complete suppression of the cell cycle drivers CDK2 and Cyclin A, leading to a promoted induction of apoptosis.

OCT is a synthetic octapeptide-based agonist for somatostatin receptors (SSTRs), which are commonly overexpressed in various types of malignancies [13]. Thus, it offers a promising tool for targeted delivery of anticancer medicines [39,40]. Herein, we investigated OCT as hitting two birds with one stone: improving the aqueous solubility of the two poorly soluble drugs while maximizing their selectivity to the cancer cells. In our novel approach, we exploited the dual solubility of OCT in both water and ethanol to solubilize the two poorly soluble drugs, PTX and DTX. The drugs in question and OCT are initially dissolved in a mixture of water and ethanol, then the organic solvent is slowly evaporated. Subsequently, the water-insoluble drugs become dispersed inside the OCT matrix, which results in the creation of a water-soluble coating of OCT around the two drugs [9]. The proposed mechanism is illustrated in Figure 8A. Compared to commercially available formulations that rely on surfactants, this novel formulation is well-tolerated in vivo, thus overcoming the biosafety concerns associated with its commercial counterparts. Moreover, its submicron particle size is beneficial in improving its absorption, bioavailability, and delivery efficiency to the tumor site [41].

First, the compatibility of the formulation components was investigated by DSC, where all individual components demonstrated characteristic endothermic peaks around their reported melting points, confirming their purity and existence in a crystalline state [42,43,44]. Upon heating PTX beyond its melting point, an exothermic peak appeared, suggesting its thermal decomposition [45]. Meanwhile, there were no peaks obtained with the OCT-PTX-DTX formulation, which suggests a substantial change in the thermal behavior of the formulation components. However, powder X-ray diffractometry (P-XRD) and complementary solid-state analyses would be required to definitively establish the physical state and molecular interactions within such a formulation [46]. Moreover, ESI-MS analysis confirmed the identity of the individual formulation components, as evidenced by m/z values corresponding to their reported molecular weights [42,43,44]. Meanwhile, the maintenance of OCT peak in the final formulation versus the disappearance of DTX peak and the transition of PTX peak suggest a potential molecular interaction between the drugs and OCT. The electron-dense aromatic rings of DTX and PTX could engage in intense π−π stacking interactions with the phenyl and indole rings of the amino acids of OCT, forming a stable, non-covalent intermolecular complex [47]. In addition, OCT possesses multiple basic nitrogen functional groups which exhibit extremely high gas-phase proton affinities during ESI. On the other hand, DTX and PTX ionize much less efficiently. Therefore, OCT might have exerted an “ion suppression” of DTX and PTX during ESI analysis [48]. Furthermore, analysis of OCT-PTX-DTX formulation by HPLC resolved and confirmed the identity of its three components. Collectively, analytical characterization verified the successful formation of the developed formulation and confirmed the successful inclusion of all of its components.

Subsequently, the physicochemical properties of the formulation were characterized, where particle size analysis revealed a bimodal particle size distribution with peaks around 0.5 µm and 1.6 µm, respectively. Such a particle size distribution is speculated to impart a sequential pattern of drug release, where an immediate drug release from smaller particles is essential to exert the initial therapeutic response, followed by a controlled pattern of drug release from the larger particles to sustain the therapeutic activity over a prolonged time period [49]. On the other hand, visualization of particles by TEM revealed a spherical morphology with a smaller diameter around 200 nm. The discrepancy in particle size obtained with TEM in comparison with light scattering-based methods may be attributed to the anhydrous conditions of sample preparation in the case of TEM, which significantly reduces the measured hydrodynamic diameter of the particles [22,50].

Next, the anticancer effects of the developed formulation were evaluated. The variability in response among the various cancerous cell lines is one of the key limitations of cell culture-based assays [51]. To cope with such a challenge, we evaluated the anticancer activities in three different human colorectal carcinoma cell lines, HT29, SW480, and SW620, to confirm the robustness of the proposed treatment. The novel formulation developed in this study showed a superior performance on the investigated cell lines compared to the free drugs, which reveals the value of such a delivery system. Moreover, the developed formulation demonstrated a higher selectivity to cancerous cells compared to a normal cell line model, MRC-5, which can be attributed to the targeting effect of OCT that promotes the affinity of the formulation to cancerous cells overexpressing SSTRs [52]. Meanwhile, OCT alone showed a negligible impact on cell viability, confirming the high tolerability of the OCT-based formulation developed herein. Furthermore, the CI values lower than 1 and the isobologram plots deviating downwards from the additive effect’s line of the two drugs are collectively evidence suggestive of synergistic interaction at the IC_50_ level. Such a synergistic effect would be beneficial in terms of reducing the therapeutic doses and the tendency of developing chemoresistance [53].

Furthermore, a cell cycle analysis performed in HT29 and SW480 colorectal carcinoma cells revealed a significant increase in the percentage of cells in the sub-G1 phase, which suggests an increase in the percentage of cells undergoing apoptosis [54]. In addition, developed formulation significantly increased the percentage of cells in the G2/M phase, which aligns with the mechanism of action of the two drugs in question. Taxanes bind to β-tubulin, a component of microtubules, disrupting the formation of the mitotic spindle, which is crucial for separating chromosomes during cell division. The damage to the mitotic spindle causes cells to become arrested in the G2/M phase of the cell cycle and induces their subsequent apoptosis [55,56]. Meanwhile, the decrease in the percentage of cells in G0/G1 and S phases supported the hypothesis of apoptosis induction in the cancer cells by OCT-PTX-DTX formulation [57,58].

Lastly, in an attempt to understand the molecular basis of OCT-PTX-DTX formulation actions, the gene expression profiles of the relevant genes in SW480 cells were mapped post-treatment. The upregulation of somatostatin receptors (SSTR1 and SSTR2) following treatment with OCT-PTX-DTX formulation suggests a possible contribution of SSTR-related signaling in the internalization of the developed formulation into the cells via receptor-mediated endocytosis, exploiting the differential expression of such receptors on the surface of cancer cells, where OCT, a pharmacological agonist of SSTRs, serves as a targeting ligand. The binding of OCT to SSTRs is thought to induce their upregulation through a positive feedback mechanism that promotes further intracellular accumulation of OCT-PTX-DTX [59]. Yet, further in-depth mechanistic investigations are needed to confirm such a hypothesis. Subsequently, the internalization of OCT-PTX-DTX nanoparticles into the cancer cells resulted in a strong induction of p53 (5.6-fold) and its downstream effector p21 (5.3-fold), which together act as a cellular barrier against genomic instability and tumor progression. Moreover, the formulation’s capacity to produce a significant cell cycle arrest is demonstrated by the striking suppression of CDK2 and Cyclin A expression (to 0.02 and 0.17-fold, respectively). Since p21 functions as a strong universal CDK inhibitor that stops the phosphorylation of proteins necessary for the G1/S transition, the near-total inhibition of CDK2 is particularly remarkable [60]. Compared to traditional, non-targeted chemotherapy, the OCT-PTX-DTX formulation offers a highly effective mechanism for treating SSTR-positive colorectal malignancies by successfully silencing these cell cycle drivers and shifting the cellular balance toward programmed cell death. Figure 8B outlines the proposed molecular mechanism of the developed formulation on colorectal cancer cells.

Collectively, the OCT-PTX-DTX formulation showed promising results in overcoming the poor physicochemical properties of PTX and DTX and rendering their maximum pharmacological effects to synergistically and selectively eradicate cancer cells at low drug concentrations and without substantial off-target effects.

## 5. Conclusions

This study provides an in vitro proof-of-concept for a novel formulation combining Paclitaxel (PTX) and Docetaxel (DTX) using Octreotide (OCT) as both a solubilizing and a targeting agent. Electrospray Ionization Mass Spectrometry (ESI-MS) and High-Performance Liquid Chromatography (HPLC) analyses confirmed the successful generation of the proposed formulation and verified the identity of its components. The developed formulation, OCT-PTX-DTX, showed a submicron particle size with the absence of any physical or chemical incompatibilities. The developed formulation demonstrated selective cytotoxicity to various cancerous cells with minimal effects on the normal cells. Moreover, the developed nanosystem induced superior anticancer activities compared to the free drugs. Furthermore, the formulation enabled the synergism between PTX and DTX for enhanced anticancer activity at lower drug concentrations. The simple composition and scalable preparation method of the OCT-PTX-DTX formulation make it promising for targeted taxane-based chemotherapy with a higher tolerability compared to the commercially available formulations.

## Data Availability

The original contributions presented in this study are included in the article/Supplementary Materials. Further inquiries can be directed to the corresponding author(s).

## Supplementary Materials

The following supporting information can be downloaded at: https://www.mdpi.com/article/10.3390/pharmaceutics18091159/s1. Figure S1: ESI-MS spectra of DTX (A), PTX (B), OCT (C), and OCT-PTX-DTX (D); Figure S2: HPLC chromatograms of PTX, DTX (A), and OCT (B) in the final OCT-PTX-DTX formulation; Table S1: The sequences of the oligonucleotide primers used in qRT-PCR experiments in this study; Table S2: Analysis of the particle size distribution of OCT-PTX-DTX formulation.
