# Supplementary material for: Octreotide-Mediated Co-Solubilization of Paclitaxel and Docetaxel: A Novel Targeted Formulation for Synergistic Anticancer Chemotherapy
Source: Pharmaceutics. 2026 Sep 15;18(9):1159. doi: 10.3390/pharmaceutics18091159 (PMC13610579; doi:10.3390/pharmaceutics18091159)
Supplement: Supplementary file 1 [file pharmaceutics-18-01159-s001.zip › pharmaceutics-4525221-supplementary.pdf]

SUPPORTING INFORMATION

Octreotide-Mediated Co-Solubilization of Paclitaxel and Docetaxel: A Novel Targeted Formulation for Synergistic Anticancer Chemotherapy

Ahmed A. H. Abdellatif [1,*], Hesham M. Tawfeek [2,3], Mahmoud Zaki El-Readi [4], Amani E. Alharbi [5], Hamzah M. Maswadeh [1], Mohammed A. Amin [1], Safaa Y. Eid [4], Abousree T. Ellethy [6], Bassem Refaat [7,†], Akhmed Aslam [7], Waleed Mohammad Altowayan [8] and Mahmoud A. Younis [2,*]

[1] Department of Pharmaceutics, College of Pharmacy, Qassim University, Qassim 51452, Saudi Arabia; msodh@qu.edu.sa (H.M.M.); m.darwish@qu.edu.sa (M.A.A.)

[2] Department of Industrial Pharmacy, Faculty of Pharmacy, Assiut University, Assiut 71526, Egypt; heshamtawfeek@aun.edu.eg

[3] Faculty of Pharmacy and Drug Research, Assiut National University, New Assiut City, Assiut 11511, Egypt

[4] Department of Biochemistry, Faculty of Medicine, Umm Al-Qura University, Al Abdeyah, Makkah 24381, Saudi Arabia; mzreadi@uqu.edu.sa (M.Z.E.-R.); syeid@uqu.edu.sa (S.Y.E.)

[5] Department of Pharmacology and Toxicology, College of Pharmacy, Taibah University, Madinah 42353, Saudi Arabia; aebharbi@taibahu.edu.sa

[6] Department of Basic Oral Sciences and Dental Education, College of Dentistry, Qassim University, Buraidah 51452, Saudi Arabia; aliethay@qu.edu.sa

[7] Department of Clinical Laboratory Sciences, Faculty of Applied Medical Sciences, Umm Al-Qura University, Al Abdeyah, P.O. Box 7607, Makkah 24381, Saudi Arabia; b.refaat@mnc.edu.sa (B.R.); maaslam@uqu.edu.sa (A.A.)

[8] Department of Pharmacy Practice, College of Pharmacy, Qassim University, Buraydah 52571, Saudi Arabia; w.altowayan@qu.edu.sa

* Correspondence: a.abdellatif@qu.edu.sa (A.A.H.A.); mahmoudyounis@aun.edu.eg (M.A.Y.)

† Current affiliation: Department of Basic Medical Sciences, Medicine Program, Makkah National College, Makkah 24231, Saudi Arabia.

SUPPORTING FIGURES

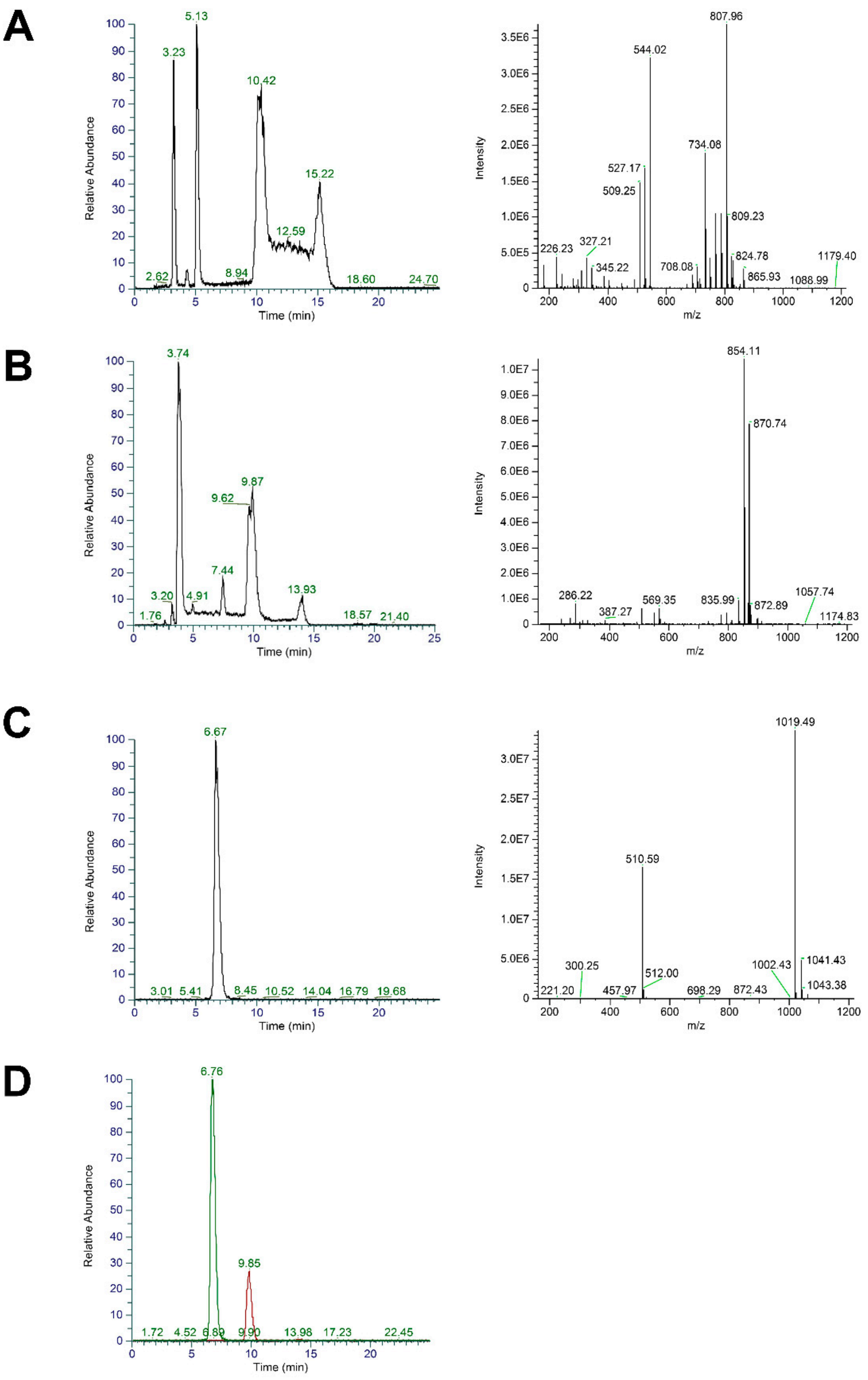

Figure S1. ESI-MS spectra of DTX (A), PTX (B), OCT (C), and OCT-PTX-DTX (D).

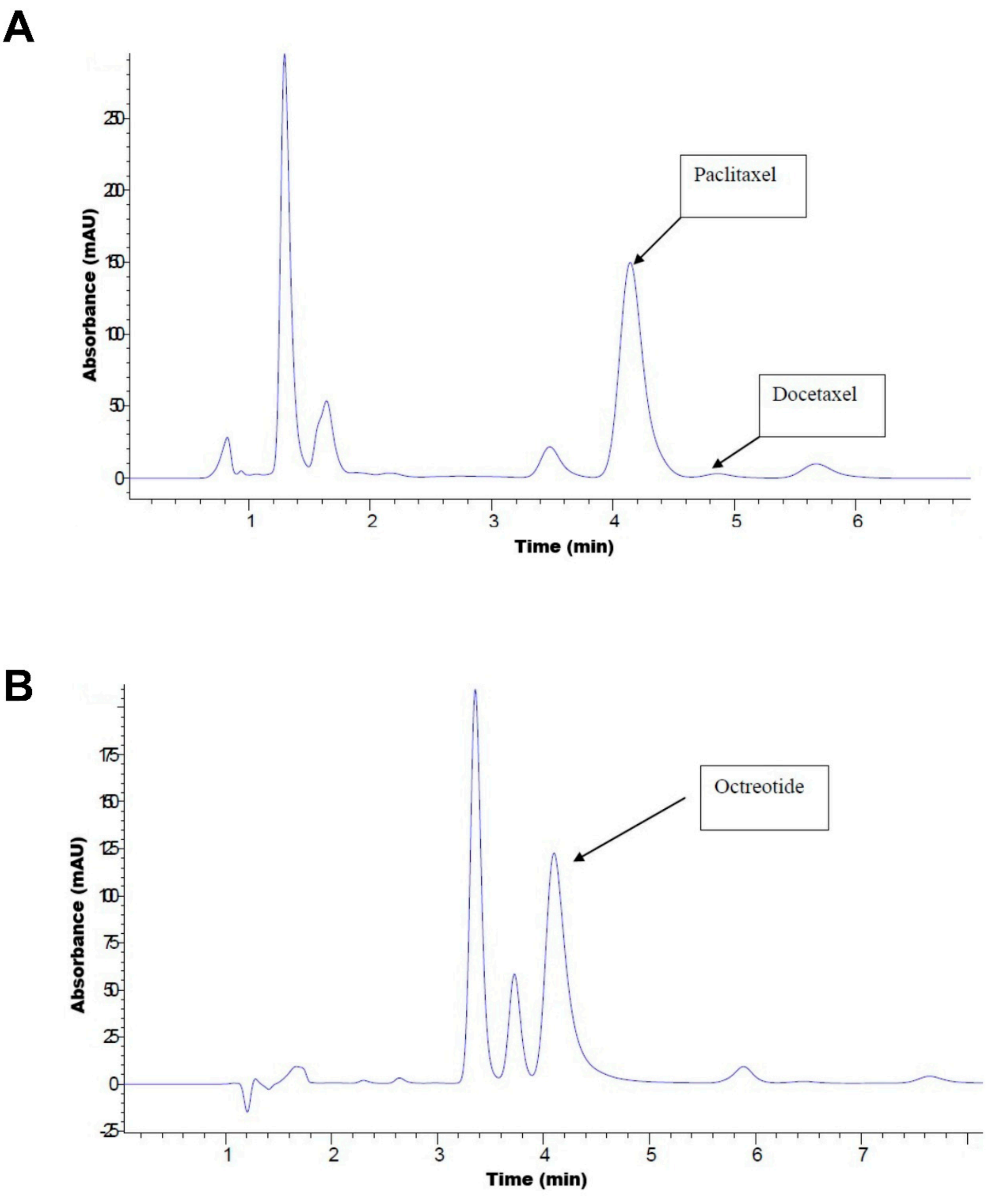

Figure S2. HPLC chromatograms of PTX, DTX (A), and OCT (B) in the final OCT-PTX-DTX formulation.

SUPPORTING TABLES

Table S1. The sequences of the oligonucleotide primers used in qRT-PCR experiments in this study.

Gene	Primer	Sequence (5' to 3')
p53 (TP53)	Forward	GAGCTGAATGAGGCCTTGGA
	Reverse	CTGAGTCAGGCCCTTCTGTCTT
p21 (CDKN1A)	Forward	TGT CCG TCA GAA CCC ATG C
	Reverse	AAA GTC GAA GTT CCA TCG CTC
CDK2	Forward	ATGGATGCCTCTGCTCTCACTG
	Reverse	CCCGATGAGAATGGCAGAAAGC
Cyclin A (CCNA2)	Forward	GCC TGG GCT CTG ATC AGA G
	Reverse	CCC CGA ACC CTT TAT TCC AGT
SSTR1	Forward	CACATTTCTCATGGGCTTCCT
	Reverse	ACAAACACCATCACCACCATC
SSTR2	Forward	GGCATGTTTGACTTTGTGGTG
	Reverse	GTCTCATTCAGCCGGGATTT
GAPDH (Internal control)	Forward	GTCTCCTCTGACTTCAACAGCG
	Reverse	ACCACCCTGTTGCTGTAGCCAA

Table S2. Analysis of the particle size distribution of OCT-PTX-DTX formulation.

Differential distribution		Cumulative distribution			
Mean D (μm)	Modal D (μm)	Median D (μm)	$D_{75\%}$ (μm)	$D_{50\%}$ (μm)	$D_{25\%}$ (μm)
0.928	0.510	0.837	1.701	0.837	0.505
